# A pilot study assessing reliability and age‐related differences in corticomuscular and intramuscular coherence in ankle dorsiflexors during walking

**DOI:** 10.14814/phy2.14378

**Published:** 2020-02-28

**Authors:** Federico Gennaro, Eling D. de Bruin

**Affiliations:** ^1^ Department of Health Sciences and Technology Institute of Human Movement Sciences and Sport ETH Zurich Zurich Switzerland; ^2^ Division of Physiotherapy Department of Neurobiology, Care Sciences and Society Karolinska Institutet Stockholm Sweden

**Keywords:** aging, corticomuscular coherence, corticospinal control, gait, intramuscular coherence, test–retest

## Abstract

Corticomuscular (CMC) and intramuscular (intraMC) coherence represent measures of corticospinal interaction. Both CMC and intraMC can be assessed during human locomotion tasks, for example, while walking. Corticospinal control of gait can deteriorate during the aging process and CMC and intraMC may represent an important monitoring means. However, it is unclear whether such assessments represent a reliable tool when performed during walking in an ecologically valid scenario and whether age‐related differences may occur. Wireless surface electroencephalography and electromyography were employed in a pilot study with young and old adults during overground walking in two separate sessions. CMC and intraMC analyses were performed in the gathered beta and lower gamma frequencies (i.e., 13–40 Hz). Significant log‐transformed coherence area was tested for intersessions test–retest reliability by determining intraclass correlation coefficient (ICC), yielding to low reliability in CMC in both younger and older adults. intraMC exclusively showed low reliability in the older adults, whereas intraMC in the younger adults revealed similar values as previously reported: test–retest reliability [ICC (95% CI): 0.44 (−0.23, 0.87); *SEM*: 0.46; MDC: 1.28; MDC%: 103; Hedge's g (95% CI): 0.54 (−0.13, 1.57)]. Significant differences between the age groups were observed in intraMC by either comparing the two groups with the first test [Hedge's g (95% CI): 1.55 (0.85, 2.15); *p*‐value: .006] or with the retest data [Hedge's g (95% CI): 2.24 (0.73, 3.70); *p*‐value: .005]. Notwithstanding the small sample size investigated, intraMC seems a moderately reliable assessment in younger adults. The further development and use of this measure in practical settings to infer corticospinal interaction in human locomotion in clinical practice is warranted and should help to refine the analysis. This necessitates involving larger sample sizes as well as including a wider number of lower limb muscles. Moreover, further research seems warranted by the observed differences in modulation mechanisms of corticospinal control of gait as ascertained by intraMC between the age groups.

## Graphical abstract

Corticomuscular and intramuscular coherence are widely used nowadays in motor control and movement neurophysiology field, but it is unclear whether these assessments represent reliable tools when performed during walking in an ecologically valid scenario. Corticospinal control of gait can deteriorate during the aging process and corticomuscular or intramuscular coherence may represent an important monitoring strategy, but whether any age‐related differences may occur still needs to be elucidated. We show that neural drive to ankle dorsiflexors during gait in an ecological valid scenario is differently modulated in aging and, in parallel, we confirm previous research showing moderate reliability of intramuscular coherence, but not yet regarding corticomuscular coherence.

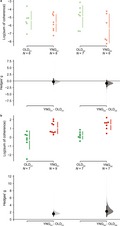

## 1 INTRODUCTION

Coherent oscillatory activity between the cortical neuronal pool and spinal motor neurons within beta and gamma frequency band has been shown to represent an effective mechanism of corticospinal interaction and central drive to skeletal muscle (Farmer, Bremner, Halliday, Rosenberg, & Stephens, 1993; Halliday, Conway, Farmer, & Rosenberg, 1998; Pfurtscheller, 1981; Schoffelen, Oostenveld, & Fries, 2005; Schoffelen, Poort, Oostenveld, & Fries, 2011). Corticospinal control of muscles can be assessed during voluntary muscle contractions either by concurrent recording of surface electroencephalography (EEG) sensors overlaying sensorimotor cortex and surface electromyography (EMG) from contralateral muscles (i.e., corticomuscular coherence; CMC) (Mima & Hallett, 1999) or by placing a pair of EMG sensors over the same muscle of interest (intramuscular coherence; intraMC) (Farina, Negro, & Dideriksen, 2014; Farmer et al., 1993). Recently, novel wearable sensor technologies and advanced procedures in signal processing fostered a great potential in assessing corticospinal control of gait during more complex human locomotion tasks, for example, during real‐world walking (Boonstra, 2013; Gennaro & de Bruin, 2018). Mounting evidence hints toward an engaged role of the central nervous system (CNS) in actively controlling human gait execution, largely investigated in the last decade within the so‐called mobile brain/body imaging (MoBI) research framework (Castermans & Duvinage, 2013; Gennaro & de Bruin, 2018; Makeig, Gramann, Jung, Sejnowski, & Poizner, 2009). CMC and intraMC have been assessed during gait in clinical settings in spinal cord injury (Barthelemy et al., 2010), in neuromuscular diseases, and movement disorders (Fisher, Zaaimi, Williams, Baker, & Baker, 2012; Larsen et al., 2017; Roeder, Boonstra, & Kerr, 2019; von Carlowitz‐Ghori et al., 2014; Willerslev‐Olsen, Petersen, Farmer, & Nielsen, 2015), but have also been applied with healthy young and older adult participants (Artoni et al., 2017; Jensen et al., 2019; Petersen, Willerslev‐Olsen, Conway, & Nielsen, 2012; Roeder, Boonstra, Smith, & Kerr, 2018; Spedden, Choi, Nielsen, & Geertsen, 2019).

Aging has been linked to adaptations of CMC (Bayram, Siemionow, & Yue, 2015), and corticospinal control of gait seems to differently modulate in older compared to younger adults (Spedden et al., 2019; Spedden, Nielsen, & Geertsen, 2018) when different gait task modalities are employed. Despite the large body of studies employing CMC and intraMC, to our knowledge, only few studies have investigated test–retest reliability of CMC (Pohja, Salenius, & Hari, 2005; Witham, Riddle, Baker, & Baker, 2011). Furthermore, solely one study assessed this during active gait, but employing only intraMC (van Asseldonk, Campfens, Verwer, Putten, & Stegeman, 2014). Studies on CMC reliability during walking seem not to be present.

Evaluating test–retest reliability when gait is actively performed in an ecological valid scenario, for example, while walking overground as opposed to laboratory‐based treadmill walking, might shed light on the clinical relevance of the assessment. Intramuscular coherence measured when walking on a treadmill has been classified as a sufficiently reliable and easy to use assessment; however, it is limited in its practical use due to the large changes needed for the confident interpretation that real change was observed rather than measurement error of intraMC (van Asseldonk et al., 2014). For clinical relevance, possible changes in corticospinal control of muscles during human locomotion should be assessed longitudinally in order to detect, for example, age‐related changes linked to neuromuscular and locomotor systems or to assess the effects of interventions aimed at restoring loss of functionality. Therefore, the aim of this study was to ascertain both test–retest reliability and age‐related differences of CMC and intraMC in ankle dorsiflexors assessed during overground walking.

## 2 MATERIAL AND METHODS

### 2.1 Participants

Eighteen healthy volunteers, nine older (three females; age: 73 ± 6 years; range: 66–84 years) and nine younger adults (five females; age: 26 ± 3 years; range: 23–31 years) were included in the study and completed all the experimental sessions. The study protocol was approved by the Cantonal Ethics Committee of Zurich (Zurich, Switzerland) and an informed consent in accordance with the Declaration of Helsinki was obtained signed by the participants. Community‐dwelling volunteers interested to participate were included if their age was from 18 to 35 years old (young group; YNG) or had an age ≥ 65 years old (old group; OLD). Moreover, the participants of this study had to fulfill the following inclusion criteria: nonsmoking in the last 12 months; body mass index (BMI) between 18.5 and 30; and Mini‐Mental State Examination score ≥22. BMI was selected as inclusion criteria because people having high BMI and, therefore, are at risk of obesity, may have gait kinematic impairments. One participant with BMI slightly above 30 was exempted from this rule (YNG group) after checking the gait kinematics. Volunteers interested to participate were excluded if they met the following criteria: self‐reporting neurological or musculoskeletal disorders impairing their sensorimotor function and/or mobility function (e.g., Parkinson's disease, dystrophy); cardiac, respiratory, liver, diabetic, renal, or psychiatric disorders impairing sensorimotor and/or mobility function; cancer treatment in the last year; inflammatory or chronic viral diseases; pharmacological treatments interfering with the electrophysiological measurements of the study or inducing muscle weakness (e.g., baclofen, muscle relaxants, etc.) or influencing the amount of inflammatory biomarkers and Omega‐3 fatty acids in the blood (e.g., inflammatory agents or Omega‐3 fatty acids supplementation); history of drug or alcohol abuse. Additionally, a geriatric depression scale (GDS) was administered but not used for inclusion/exclusion criteria or further analyzed in the present study.

### 2.2 Experimental protocol

Experimental measurements were performed on two nonconsecutive days with 48 hr in‐between sessions. When possible, each participant took part at each session at the same time of the day. The figure‐8 gait path was structured by two custom‐built, parallel pipe‐shaped structures with an in‐between distance of ~5 m. Participants were asked to walk continuously without stopping by turning around each of these two structures. On top of each structure, a big easy‐to‐spot arrow was placed to indicate the direction and side of turning. Participants started the gait trial from one of these two structures, which was always kept the same, and whenever they were comfortable and ready to start. Start was always after a verbal “start” call and subjects were expected to walk continuously until a subsequent “stop” call was verbally expressed by the experimenter. Participants were asked to walk at a self‐selected preferred walking speed. The gait trial was considered completed when the participant performed a total of 30 figure‐8 loops. Counting of figure‐8 gait loops was performed by the experimenter and not by the participant in order to avoid any possible dual‐task cognitive additional load. A tape was applied on the ground at ~1 m distance from the structure to manually trigger the beginning and ending of straight walking parts of the gait path by manually pressing specific computer keyboard keys. Participants were instructed to walk naturally as soon as possible to maintain ecological validity of the experimental protocol but, at the same time, they were asked to maintain their gaze straight toward the arrow placed on top of each structure in front of them as much as possible during the straight part of the gait trial. When walking the curved part of the gait path, no instruction relative to the gaze was provided. Before executing the gait trial, participants were asked to perform a familiarization walking trial of ~5 min as warm up. Before all the gait trials were executed, a weak isometric muscle contraction trial (i.e., 10% MVC) of ~120 s with both right and left ankle dorsiflexors was performed, where the reference value was based on the three previously executed maximum voluntary contraction (MVC) trials of ~5 s (per side and with ~120 s in‐between rest). Participants were asked to perform ankle dorsiflexion trials using biofeedback from the EMG vendor‐provided software, and against the hand of the (always the same) experimenter opposing the movement and providing standardized verbal encouragement during the test.

### 2.3 Data acquisition

Surface EEG activity was recorded at a sampling frequency of 500 Hz by a high‐density 64‐channel EEG system (*eego sport*, ANT Neuro, Enschede, The Netherlands). Three EEG cap sizes were employed in order to accommodate different head circumferences (*waveguard*, ANT Neuro) and EEG electrodes were placed according the 10–10 international system (Chatrian, Lettich, & Nelson, 1985). An additional electrooculography (EOG) electrode was placed below the left eye at the level of the inferior part of the orbital fossa. EEG was referenced to CPz and ground electrode was placed at the midpoint of the right collar bone. An electrode impedance ≤10 kΩ was required before commencing measurements and it was checked throughout the progression of the experiment. Surface EMG activity was recorded at a sampling frequency of 1,500 Hz (*DTS TeleMyo*, Noraxon) by means of two pairs of bipolar Ag‐AgCl electrodes (Ambu Blue Sensor N, Ambu A/S). One of the two pairs of bipolar electrodes was placed over the left tibialis anterior (TA) muscle, while the other was over the right TA muscle. In each pair, one sensor was placed proximally (TAprox) while the other was placed distally (TAdist) with respect to the muscle belly and according to previously described anatomical landmarks (de Bruin, Patt, Ringli, & Gennaro, 2019; Jensen et al., 2019, 2018; Spedden et al., 2019; van Asseldonk et al., 2014). The interelectrode distance (electrodes’ center‐to‐center) was set to ~ 2 cm and the distance between bipolar configurations in each leg was ∼11 cm (σ: ∼2 cm; range: ∼7 cm to ∼14 cm), to reduce the risk of cross‐talk as well as the recording of muscle activity from overlapping motor unit areas (Hansen et al., 2005). The skin was properly cleaned and, when necessary, shaved before placing the EMG electrodes. Heel strike onsets were detected by placing two foot switches approximately on the midpoint of the calcaneus in both feet. EEG and EMG recordings were synchronized by sending an analog square wave pulse to both EEG and EMG system from a custom‐made device equipped with Transistor–Transistor logic (TTL) ports in order to align both time series in the subsequent data analysis.

### 2.4 Data analysis

**Figure 1:**
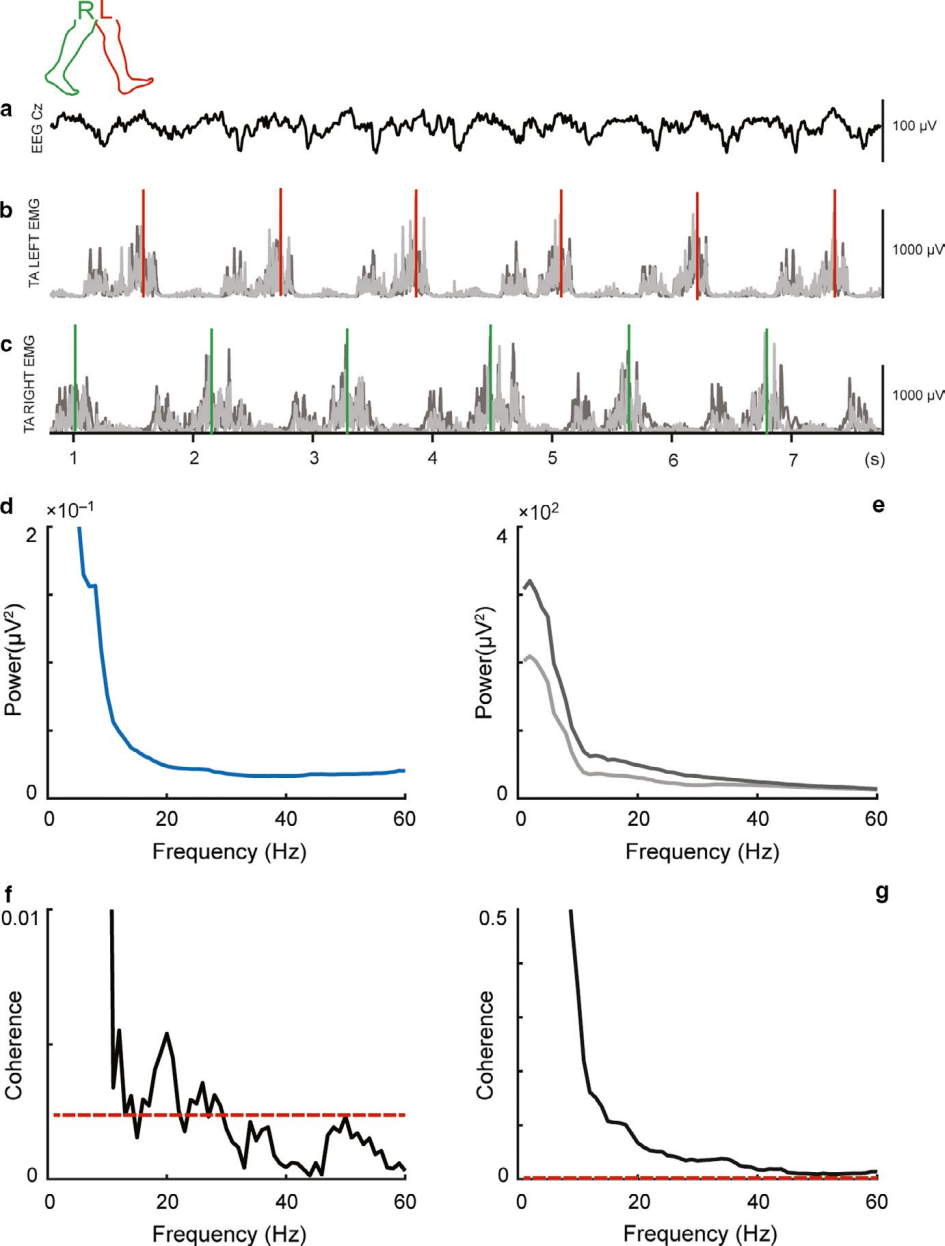
Representative data from a participant showing concurrently recorded signals during gait. In the panel, (a) shows raw filtered EEG activity at Cz electrode, while raw filtered and rectified EMG activity from proximal (light greyish color) and distal (dark greyish color) sensors placed over left and right tibialis anterior muscles are shown in the panels (b and c), respectively. All physiological signals were aligned to right (greenish color) and left (reddish color) heel‐strike events. EEG power spectrum is depicted in panel (d), while proximal and distal EMG are depicted in panel (e). In panel (f) is depicted magnitude‐squared corticomuscular coherence between EEG and (proximal) EMG, whereas in panel (g) is depicted intramuscular coherence between proximal and distal EMG is depicted

All signal processing was performed using custom‐made scripts and Fieldtrip, an open‐source toolbox for electrophysiological data analysis (Oostenveld, Fries, Maris, & Schoffelen, 2011) for Matlab (Mathworks Inc.). After alignment of the EMG data according to the TTL pulse, EMG data were downsampled to 500 Hz, in order to match the EEG sampling rate, and concurrently demeaned as well as detrended. Afterward, EMG data were high‐pass filtered (two‐pass Butterworth filter, fourth order, 20 Hz cutoff) and powerline noise as well as its harmonics were filtered out using a notch filter based on discrete Fourier transformation (DFT). Filtered EMG data were then full‐wave rectified using the Hilbert transform as a widely used preprocessing step before undertaking further coherence analysis (Boonstra & Breakspear, 2012). EEG data were aligned according to the TTL pulse and, therefore, to the EMG data. Only straight parts of the figure‐8 gait path were further considered for analysis of the aligned EEG/EMG data. After removing mastoid and ocular electrodes from further analysis (M1, M2, and EOG), EEG data were then band‐pass filtered (two‐pass Blackman‐windowed sync filter, 2,752 order, 1.5–48 Hz cutoff) and concurrently demeaned as well as detrended. Powerline noise and harmonics were filtered out as described above. Noisy channels were detected and removed if they were flat for >5 s or the correlation between neighboring channels was <0.4. Further data analysis was not performed if the total amount of removed channels was higher than half of the total scalp electrodes. On average, ~10 channels were removed (σ: ∼6; range: 2–23). The analysis of two OLD participants (one in D1 and one in D2) was not performed given that the number of rejected channels was too high. A nonstationary method was employed to clean the occasionally large amplitude noise and increase the stationarity of EEG data in preparation of the next independent component analysis (ICA) cleaning step. For this purpose, a sliding window adaptive approach based on principal component analysis (PCA) decomposition was used by means of the Riemannian modified version of the Artifact Subspace Reconstruction (rASR) method (Blum, Jacobsen, Bleichner, & Debener, 2019). The entire data were used as calibration data and a lax threshold was chosen as parameter (20 standard deviations), as previously recommended, to be large enough to reduce artifactual activity from EEG data while preserving cerebral activity (Artoni et al., 2017; Chang, Hsu, Pion‐Tonachini, & Jung, 2019). The combined use of ICA and Artifact Subspace Reconstruction has been suggested to represent an effective strategy to remove artifactual signals from EEG data (Pion‐Tonachini, Hsu, Chang, Jung, & Makeig, 2018) and it has been largely used in studies involving cleaning of EEG data acquired during human locomotion tasks such as gait (Arad, Bartsch, Kantelhardt, & Plotnik, 2018; Artoni et al., 2017; Bulea, Kim, Damiano, Stanley, & Park, 2015; Kline, Huang, Snyder, & Ferris, 2015; Nathan & Contreras‐Vidal, 2015; Peterson & Ferris, 2019). Portions of data not completely repaired by rASR were removed if more than 30% of channels were noisy in that data segment. Previously rejected noisy channels were then interpolated using spline interpolation and afterward, EEG data were re‐referenced to an average reference and then EEG signals were decomposed into temporally maximally independent components (ICs) by applying the remaining rank of the data Adaptive Mixture ICA (AMICA) with enabled online artifacts rejection using a threshold of five standard deviations in five iterations intervals starting after the first five iterations and the whole procedure repeated five times. AMICA algorithm was chosen given that it has been shown to outperform other ICA algorithms (Delorme, Palmer, Onton, Oostenveld, & Makeig, 2012). After AMICA, a machine learning‐based approach was used to identify cerebral ICs by employing the ICLabel classifier (Pion‐Tonachini, Kreutz‐Delgado, & Makeig, 2019). Two participants (one in OLD and one in YNG group, both in the D2) were not considered for further analysis because the total number of retained brain ICs was too low. On average, ~11 cerebral ICs (σ: ∼5; range: 4–21) were identified by ICLabel. The respective ICA weights and sphere matrices of the retained cerebral ICs were conveyed to an EEG dataset identical but processed using a more conventional filtering approach (high‐pass filter: two‐pass Hamming‐windowed sync filter, 3,300 order, cutoff 0.5 Hz; powerline noise filtered as in the EMG analysis described above). EEG and EMG data related to the isometric 10% MVC trials were aligned and segmented from EMG onset to ~120 s and then analyzed using the same preprocessing parameters mentioned above, and by segmenting again each trial in nonoverlapping epochs of ~1 s. Figure 1 shows the main data analysis steps, including the further spectral and connectivity analyses.

### 2.5 Spectral and connectivity analyses

In the present study, we have chosen to focus on the EEG Cz electrode for further spectral analysis of CMC. This vertex‐located sensor is widely employed to assess CMC during gait as well as during isometric contraction tasks using lower limbs’ muscle, such as ankle dorsiflexors (Jensen et al., 2019, 2018; Petersen et al., 2012; Ritterband‐Rosenbaum et al., 2017; Spedden et al., 2019, 2018; Yoshida, Masani, Zabjek, Chen, & Popovic, 2017).

The cleaned preprocessed EEG and EMG data were then segmented according to the swing phase from 650‐ms to 50‐ms before heel strike onsets in analogy to previous studies (Jensen et al., 2019, 2018; Petersen et al., 2012; Spedden et al., 2019), avoiding the inclusion of any potentially remaining artifact due to the collision of the foot with the ground. Two participants (one in OLD and one in YNG, in the D1 and D2, respectively) were not considered for further analysis because the total amount of trials (i.e., heel strikes) was excessively low, and given that at least 25 trials per participant were used as minimum threshold criterion for further analysis, consistent with literature (de Bruin et al., 2019). On average, ~231 gait segments (σ: ∼109; range: 39–434, considering the sum of left and right heel strikes) were used for coherence estimation. The heel‐strike segments from one participant of OLD at D1 were further analyzed only from one side, given that heel strikes from the other side were not obtained most likely due to technical problems. Data segments were zero‐padded up to 2 s and tapered with a variable set of discrete prolate spheroidal (Slepian) sequences by applying a multi‐taper frequency transform yielding a broad 1–60 Hz frequency band power‐ and cross‐spectra with a frequency resolution set to 1 Hz. Given that this analysis focused almost entirely on the beta frequency band (i.e., 13–35 Hz), with a small portion of lower gamma frequencies (i.e., 36–40 Hz), we sought to adopt eight tapers resulting in a spectral smoothing of ±7.5 Hz. With this strategy, we assured to encompass the frequencies of interest (FOI) for this study using a total bandwidth of ~15 Hz and, therefore, including the entire beta frequency band, where the bandwidth is usually found to be ∼10 Hz, but also partially the gamma frequency band, where the bandwidth is reported to be ∼25 Hz (Schoffelen et al., 2011). The following equation was used to calculate power‐ and cross‐spectra:

(1) $$S_{\mathrm{xy}}\left(f\right)=F_{x}\left(f\right)\times F_{y}{\left(f\right)}^{*}$$

where $F_{x}\left(f\right)$ or $F_{y}\left(f\right)$ denotes the Fourier transform of the signal *x* (or *y*) relative to the frequency *f* and ^∗^ denotes the complex conjugate. In this analysis, signal *x* represents either TAprox or Cz, whereas signal *y* represents TAdist or TAprox, for intraMC (TAprox‐TAdist) or CMC (Cz‐TAprox), respectively. When *x ≠ y*, $S_{xy}\left(f\right)$ denotes the cross‐spectra between signal *x* and signal *y*, relative to the frequency *f*. When *x = y*, $S_{xy}\left(f\right)$ is reduced to $S_{xx}\left(f\right)$ or $S_{yy}\left(f\right)$, which consists of the (auto) power spectra of the signal *x* (or *y*), relative to the frequency *f*. Single segments of power‐ and cross‐spectra obtained after averaging across tapers were employed to estimate coherence relative to intraMC or CMC, with the following equation:

(2) $${\mathrm{Coh}}_{\mathrm{xy}}=\frac{\left|〈S_{\mathrm{xy}}〉\right|}{\sqrt{〈S_{\mathrm{xx}}〉\times 〈S_{\mathrm{yy}}〉}}$$

where ⟨⋅⟩ denotes the yielded power‐ or cross‐spectra after averaging across data segments. Coherence is a spectral measure representing the linear correlation between signal *x* and signal *y*, where the estimate ranges between 0 and 1, with 0 representing no linear association and 1 perfect relation at a specific frequency *f*. The obtained coherence was transformed to magnitude‐squared coherence by squaring the coherence estimation and it was considered significant if it exceeded a confidence limit (CL) with a probability of 95% (α = 0.05) and related to the number of segments used for the calculation of the coherence estimate by the following equation (Rosenberg, Amjad, Breeze, Brillinger, & Halliday, 1989):

(3) $$\mathrm{CL}=1-\alpha^{1/\left(N-1\right)}$$

where *N* denotes the number of segments, represented by the number of heel strikes multiplied by the number of tapers used in the multi‐tapered spectral analysis. Although either a standard consensus or common practice in coherence analysis pipelines and procedures is lacking, a statistical test to infer on significant coherence estimates can be useful to control the inherent variability in coherence estimates related to the number of segments (e.g., number of heel strikes) used in the analysis (van Asseldonk et al., 2014) as well as avoiding spurious coherence values (Rosenberg et al., 1989). This method is widely used in literature on corticospinal control of gait (Jensen et al., 2019, 2018; Petersen et al., 2012; Spedden et al., 2019, 2018) as well as in studies on CMC using motor/low‐level force tasks (Chakarov et al., 2009; Kristeva, Patino, & Omlor, 2007; Omlor, Patino, Hepp‐Reymond, & Kristeva, 2007). Differently, one may opt to perform Fisher Z‐transformation and nonparametric permutations statistical testing (de Bruin et al., 2019; Maris, Schoffelen, & Fries, 2007), which is an alternative valid method to overcome the inconsistent number of segments used for the coherence analysis. However, in this study, we have chosen the first option given that it gives the possibility to exclude nonsignificant (and possibly not spurious) coherence estimates also at subject‐level and not exclusively at group‐level (as the second method would do). Hence, the first method has the potential to be translated in a clinical context where it is important to perform analysis on individual subject‐level (e.g., a single patient). CMC was estimated for the left and right side separately, to take into account the unequal number of segments between sides and per participant. However, for further statistical analysis, the maximum CMC estimate between left and right side was used, and, in case only one side was used for spectral analysis (i.e., because of technical problems in a specific foot switch and side), then only one side was considered for further analysis.

In the present study, we focused on the collated FOI from 13 to 40 Hz, given that the central drive to muscles during gait has been shown to be largely present in this gathered frequency band (Barthelemy et al., 2010; de Bruin et al., 2019; Jensen et al., 2019, 2018; Kitatani et al., 2016; Norton & Gorassini, 2006; Petersen et al., 2012; Spedden et al., 2019).

In our small cohort of participants and in agreement with previous studies (Fisher et al., 2012; Ushiyama et al., 2011), a significant coherence was not consistently found in the 10% MVC trials, as opposed to the gait trials where a significant coherence was found in almost all participants. Therefore, given that the aim of the study focused primarily on CMC and intraMC during gait, while isometric muscle contraction trials served for additional comparison purposes, we do not consider CMC and intraMC data related to 10% MVC trials further. Moreover, when performing the analyses that led to our manuscript, we also analyzed the study volunteers as one single group, given the small sample size involved in this study. Because we found no results that largely deviated from the separate group analyses and our focus was comparing young against older individuals, we decided these grouped results redundant, and, hence these additional results will not be discussed further in the present study.

### 2.6 Statistics

In order to ascertain reliability of CMC and intraMC measurements, single‐score absolute agreement was estimated by means of intraclass correlation coefficient [ICC (2,1)], employed using two‐way random effects analysis of variance (ANOVA) and by portioning observed total variance of the coherence estimations into variance between subjects (${MS}_{S}$), variance between sessions (${MS}_{T}$), and error variance (${MS}_{E}$), using the following formula:

(4) $$ICC\left(2,1\right)=\frac{{\mathrm{MS}}_{S}-{\mathrm{MS}}_{E}}{{\mathrm{MS}}_{S+}{\mathrm{MS}}_{E}\left(k-1\right)+\frac{k}{n}\left({\mathrm{MS}}_{T}-{\mathrm{MS}}_{E}\right)}$$

where *n* denotes the number of subjects and *k* the number of raters (or testing sessions). Missing data in either testing session (e.g., because significant coherence was not found in that subject/testing session) have been handled with listwise deletion of that subject data of both testing sessions. In addition, standard error of measurement (*SEM*), which denotes the extent of true value distribution between repeated measurements with the assumption that no systematic errors are present, was calculated with the following equation:

(5) $$SEM=\sqrt{{\mathrm{MS}}_{E}}$$

where ${MS}_{E}$, as above, represents error variance. Moreover, minimal detectable change (MDC), which represents the smallest change needed to exceed the measurement error of the repeated measurements, was also calculated at a confidence interval of 95%, with the following formula:

(6) $$\mathrm{MDC}=\mathrm{SEM}*\sqrt{2}*1.96$$

MDC was expressed, in addition, as a percentage of MDC (%MDC). We anticipate that for some variables, given the small sample size of the present study, together with a likely lower between subjects’ variance compared to within‐testing sessions variance, the resulting ICC estimates would be rather low. Therefore, we decided to explore, additionally, the effect size of between‐testing session comparisons. This has been performed using Data Analysis with Bootstrapped ESTimation (DABEST), a data analysis strategy which uses estimation statistics (Ho, Tumkaya, Aryal, Choi, & Claridge‐Chang, 2019). Estimation statistics is considered a superior statistics compared to dichotomous significance testing, focusing on effect sizes and relative precision (Ho et al., 2019). P‐values are reported along with the corresponding effect size and confidence interval (CI). Estimation of the 95% CI mean difference was calculated by performing 5,000 bootstrapping resamples. For the between testing session comparisons, a nonparametric Wilcoxon test was used within the DABEST framework. Furthermore, comparisons between OLD and YNG were performed using the abovementioned DABEST approach and performing the same amount (i.e., 5,000) of bootstrapping resamples for the 95% CI, however, using the nonparametric Mann‐Whitney test. In all DABEST nonparametric comparisons, the magnitude of the effect was calculated as Hedge's *g*, which is similar to Cohen's *d*, but corrected for small sample bias. As in Cohen's *d*, an effect size ≥0.2 was considered small, an effect size ≥0.5 medium, and an effect size ≥0.8 was considered large.

## 3 RESULTS

**Figure 2:**
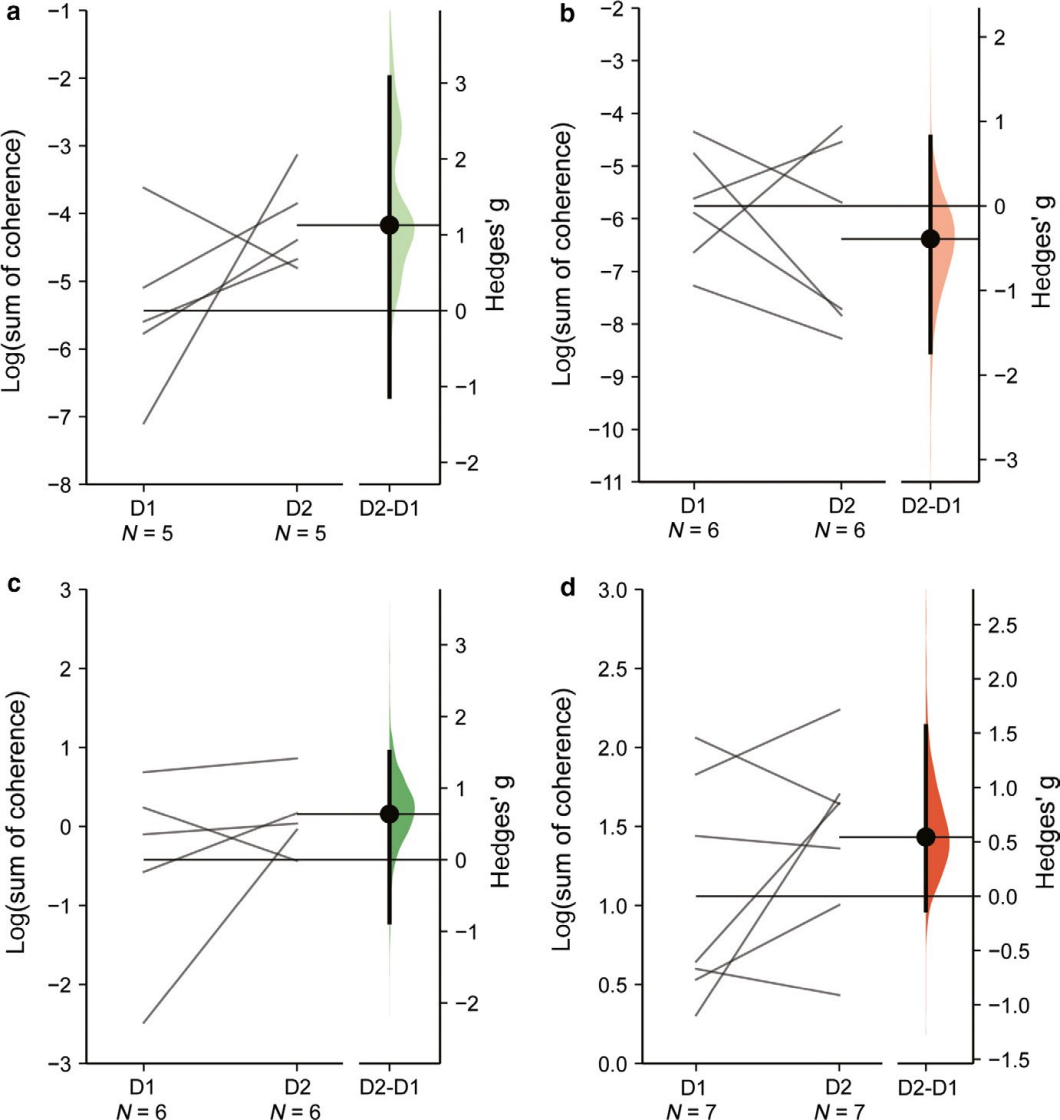
Gardner‐Altman estimation plots showing mean differences of the log‐transformed coherence area (sum of coherence above significant confidence limits) relative to CMC in the older adult (a) and in the younger adult groups (b) as well as intraMC in the older adult (c) and younger adult groups (d). In each plot, both testing sessions (day 1 and day 2, D1 and D2, respectively) are plotted on the left axes as a slopegraph and connected by a line. In each plot, the paired mean difference is plotted on the floating axes on the right as a bootstrap sampling distribution. Mean differences are represented by dots and the ends of the vertical error bars denote the 95% confidence intervals

Figure 2 presents the results of the reliability analysis (listed in Table 1) employing ICC together with *SEM*, MDC, and %MDC, which lead to low values in CMC estimates in younger adults [ICC(95% CI): −0.44 (−0.64, 0.49), *SEM*: 1.29, MDC: 3.58, %MDC: −75] as well as in the older adults [ICC (95% CI): 0.09 (−0.80, 0.80), *SEM*: 1.41, MDC: 3.92, %MDC: −65], respectively. Figure 2 also presents statistical comparisons and effect size estimations as performed with DABEST. Effect sizes were variable ranging from small to large and were not significant when comparison of CMC between measurement days was taken into account [Hedge's g (95% CI): 1.13 (−1.14, 3.08), *p* = .138 and Hedge's g (95% CI): −0.39 (−1.73, 0.82), *p* = .463, respectively]. When intraMC was taken into account, low reliability was observed in older adults [ICC (95% CI): 0.26 (−0.50, 0.84), *SEM*: 0.74, MDC: 2.05, %MDC: −3247], while in younger adults, the estimate was fairly reliable [ICC (95% CI): 0.44 (−0.23, 0.87), *SEM*: 0.46, MDC: 1.28, %MDC: 103]. In both cases, effect sizes from DABEST were medium [Hedge's g (95% CI): 0.64 (−0.87, 1.51), *p* = .249 and Hedge's g (95% CI): 0.54 (−0.13, 1.57), *p* = .237, in older and younger adults, respectively].

**Table 1:** Test–retest reliability estimates and DABEST comparison with effect sizes and respective p‐values

	ICC (95% CI)	*SEM*	MDC	MDC%	Hedge's g (95% CI)	*p*‐value
CMC_old_	−0.44 (−0.64, 0.49)	1.29	3.58	−75	1.13 (−1.14, 3.08)	.138
CMC_yng_	0.09 (−0.80, 0.80)	1.41	3.92	−65	−0.39 (−1.73, 0.82)	.463
intraMC_old_	0.26 (−0.50, 0.84)	0.74	2.05	−3247	0.64 (−0.87, 1.51)	.249
intraMC_yng_	0.44 (−0.23, 0.87)	0.46	1.28	103	0.54 (−0.13, 1.57)	.237

Abbreviations: CI, confidence interval; CMC, corticomuscular coherence; ICC, intraclass correlation coefficient; intraMC, intramuscular coherence; MDC, minimum detectable changes; *SEM*, standard error of measurements.

**Figure 3:**
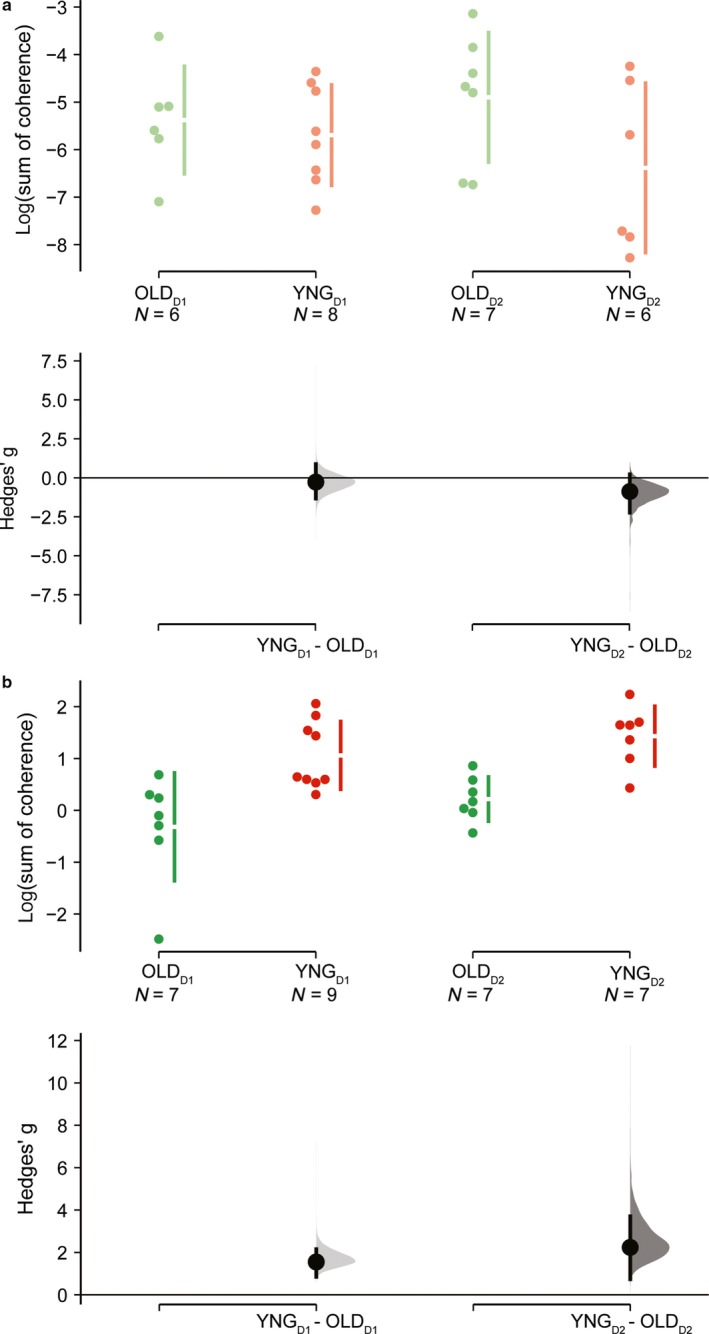
Cumming estimation plots showing mean differences of the log‐transformed coherence area (sum of coherence above significant confidence limits) relative to CMC (a) and intraMC (b). In each plot, raw log‐transformed coherence area is plotted in the upper axes for both older adult (OLD, reddish color) and younger adult (YNG, greenish color) groups and separately for each measurement days (day 1 and day 2, D1 and D2, respectively). Each mean difference is represented by dots and plotted on the lower axes as a bootstrap sampling distribution and the ends of the vertical error bars denote the 95% confidence intervals

In Figure 3, statistical comparisons between age groups and magnitude of effects from DABEST analysis are depicted (listed in Table 2). When CMC was compared between age groups, the effect size was small to large but always nonsignificant in both day 1 [Hedge's g (95% CI): −0.27 (−1.33, 0.88), *p* = .65] and day 2 [Hedge's g (95% CI): −0.88 (−2.24, 0.22), *p* = .175]. In contrast, when intraMC was taken into account, the magnitude of effect was large and significant at both day 1 [Hedge's g (95% CI): 1.55 (0.85, 2.15), *p* = .006] and day 2 [Hedge's g (95% CI): 2.24 (0.73, 3.70), *p* = .005].

**Table 2:** DABEST comparison with effect sizes and respective p‐value at both D1 and D2 testing session

	Hedge's g (95% CI)	*p*‐value
CMC_old_ versus CMC_yng_ @Day 1	−0.27 (−1.33, 0.88)	.651
CMC_old_ versus CMC_yng_ @Day 2	−0.88 (−2.24, 0.22)	.175
intraMC_old_ versus intraMC_yng_ @Day 1	1.55 (0.85, 2.15)	.006
intraMC_old_ versus intraMC_yng_ @Day 2	2.24 (0.73, 3.70)	.005

Abbreviations: CI, confidence interval; CMC, corticomuscular coherence; intraMC, intramuscular coherence.

## 4 DISCUSSION

This study aimed to explore whether CMC and intraMC can represent a reliable assessment of corticospinal control assessed during overground walking in differently aged populations. Furthermore, the study investigated whether different corticospinal control of gait modulations can be linked to these two age groups. Our results show that, at the current state of the art of measurement and in older adults, neither CMC nor intraMC represents a reliable measurement of corticospinal control of gait in older adults, provided that tibialis anterior muscle and swing phase of gait are taken into account. The same was observed for intraMC in the older adult group, whereas in young adults, intraMC showed fair reliability. This latter result matches closely with the previous results of intraMC (with regard to the magnitude of reliability and agreement estimates), which considered the same lower limb muscle (i.e., tibialis anterior) with younger adults but during gait performed on a treadmill (van Asseldonk et al., 2014). This indicates that intraMC has potential in experimental contexts aimed at exploring corticospinal control of gait dynamics provided that young adults and tibialis anterior muscles are the targets of an intervention and that swing phase of gait is the focus of this involved locomotor task. Indeed, the present study explored corticospinal control of gait only during the swing phase of gait, to be in line with previous studies investigating the same gait phase (de Bruin et al., 2019; Petersen et al., 2012; van Asseldonk et al., 2014), however, it remains unclear whether different gait phases will show comparable results. Indeed, promising investigations in this direction have been reported and, in particular, the double limbs support phase of walking showed to differently modulate corticospinal control of gait (Artoni et al., 2017; Roeder et al., 2019, 2018). Moreover, future studies could consider exploring more muscles than only tibialis anterior.

Regarding the observed low reliability results observed in our study, we need to emphasize that this finding should be interpreted with caution. Indeed, when ICC estimates are low or even negative as in our case (i.e., as observed in CMC of older adults), this may very well be due to the small sample size, which might be responsible for the lack of variance between participants of the study, which may have impaired the statistical reliability results. However, this should not stop researchers from further exploring the use of EEG technology in conditions where locomotor tasks are involved (Artoni et al., 2017). When comparing intraMC between young and older adults (in both testing sessions), higher log‐transformed coherence area values in young compared to older adults were observed. This observed significant difference, coupled with the large magnitude of effect, might be explained by the possibility that older adults can have a reduced neural drive to their muscles, at least during the swing phase of the gait cycle and in regard to tibialis anterior. An impaired neural drive to muscles may have important implications for future studies aimed to explore muscle functionality in older adults, because, for example, reduced neural drive to muscles is associated with reduced muscle strength production capacity Clark & Taylor, 2011; Tieland, Trouwborst, & Clark, 2018).

Sample size was not the sole limitation of this study, indeed, also considering more lower limbs muscle other than tibialis anterior muscles only can be useful in exploring whether different muscles may have similar or different reliability patterns and/or differently modulating connectivity with EEG; e.g. the quadriceps muscle (Roos, Rice, Connelly, & Vandervoort, 1999). Another limitation may be the fact that EEG data was not completely denoised from movement‐related artifacts. However, it should be noted that it is almost impossible to remove all artifacts/noise present in EEG data, and that we adopted extensive care to denoise our EEG data, for instance by not considering data segments subsequent of heel strikes, which might have been excessively contaminated by artifacts due to the collision of the foot with the ground. We also applied ASR and multimodal AMICA algorithms as well as machine learning techniques for detecting artifactual/cerebral ICs components (i.e., ICLabel), and, in addition, adopted the strategy of transferring ICA weights and spheres to a more conventional non‐ASR cleaned dataset. The latter because this may have impaired the subsequent connectivity analysis (Artoni et al., 2017).

## 5 CONCLUSIONS

Due to the exploratory nature of this study, we cannot firmly state that neither CMC nor intraMC are reliable methods of assessment. However, provided that tibialis anterior is assessed during the swing phase of overground walking in young adults, we can confirm that intraMC is a fairly reliable method to assess corticospinal control of gait in this specific age group using the current state of the art ways of analysis. CMC currently does not, yet, represent a satisfactorily reliable assessment of corticospinal control of gait. Our finding from the comparison between the two age groups seems to hint toward a reduced neural drive to muscle in older adults, a finding that warrants further exploration of the assessment, representing an important starting point for future studies seeking to explore age‐related differences in motor control tasks (i.e., gait) possibly linked to muscle functionality (i.e., muscle strength/mass). Further multidisciplinary research, involving bigger sample sizes and considering more muscles for connectivity analysis, is needed.

## CONFLICT OF INTEREST

The authors declare no conflict of interest.

## AUTHOR CONTRIBUTIONS

FG developed the research question, conceptualization, methodology, and design of the study, while EdB acted as methodological council. FG conducted data acquisition, analysis, and interpretation of the results with editing and improvement by EdB. FG produced a first original version of the manuscript, while EdB revised and edited the manuscript to bring it to its current version. Both authors have read and approved the final manuscript.
